# Cervical mucus proteomics approach for swamp buffalo secreted fertility biomarker identification

**DOI:** 10.2478/jvetres-2026-0025

**Published:** 2026-05-14

**Authors:** Muhammad Yusuf, Abdul Latief Toleng, Hasrin Hasrin, Abdullah Baharun, Athhar Manabi Diansyah, Ekayanti Mulyati Kaiin, Masturi Masturi, Sahiruddin Sahiruddin, Miftahul Jannah

**Affiliations:** 1 Animal Production, Faculty of Animal Husbandry, Animal Production and Technology, Faculty of Vocation, Hasanuddin University, Makassar 90245, Indonesia; 2 Animal Production and Technology, Faculty of Vocation, Hasanuddin University, Makassar 90245, Indonesia; 3 Animal Science, Faculty of Agriculture, Djuanda University, Ciawi 16720, Indonesia; 4 Research Center for Applied Zoology, National Research and Innovation Agency, Bogor 16911, Indonesia; 5 Master Program of Animal Biomedical Sciences, School of Veterinary Medicine and Biomedical Sciences, IPB University, Bogor 16680, Indonesia

**Keywords:** artificial insemination timing, decision support, luminal secretions, mucosal proteome, swamp buffalo

## Abstract

**Introduction:**

Reproductive efficiency in swamp buffalo (*Bubalus bubalis*) remains constrained by the lack of molecular tools to identify fertile females before service. The aim of this study was to discover biologically grounded biomarkers of fertility.

**Material and Methods:**

Cervical mucus was profiled using high-resolution proteomic analysis, with quantitative and functional enrichment used to prioritise biologically relevant fertility markers.

**Results:**

The cervical-mucus proteome exhibited a conserved secreted core with marked quantitative shifts that separated favourable-quality from poor-quality mucus. Favourable mucus was characterised by a barrier- and adhesion-associated profile with antimicrobial and antioxidant features, its proteins including integrin/extracellular matrix interactors, redox enzymes, lipocalin 2 (LCN2), secretory leukocyte protease inhibitor (SLPI) and heat-shock protein B6 (HSPB6). Poor mucus showed a neutrophil–acute-phase axis with complement engagement and oxidative chemistry, typified by myeloperoxidase (MPO), pentraxin 3 (PTX3) and C-reactive protein (CRP). Functional enrichment emphasised extracellular and host-defence processes consistent with these opposing mucosal states. These six proteins were consistently the leading discriminants and had coherent biological roles.

**Conclusion:**

Swamp-buffalo cervical mucus is variously in a barrier-competent state and a neutrophil-dominant state. This dichotomy provides a mechanistic explanation for fertility differences at the cervix and yields actionable, secreted protein biomarkers – LCN2/SLPI/HSPB6 in favourable mucus and MPO/PTX3/CRP in poor mucus – for targeted assays and pre-service screening in tropical production systems.

## Introduction

Reproductive efficiency is a principal driver of livestock productivity in Southeast Asia, particularly for the swamp buffalo (*Bubalus bubalis*), which underpins smallholder livelihoods and contributes to national protein security initiatives in Indonesia (18). Despite these animals’ resilience and adaptability to humid–tropical systems, field fertility outcomes in buffalo often fall short of programmatic targets for genetic progress, with artificial insemination (AI) performance constrained by heterogeneous management, heat-detection challenges and underuse of physiology-informed selection tools (4, 5). These limitations highlight the need for robust, biology-anchored criteria to identify females with high conception potential before service.

A central barrier is the paucity of molecularly guided selection strategies for female fertility. Current decision-making in many buffalo programmes remains dominated by coarse phenotypes (*e.g*. age, body condition and calving interval) that only partially capture the complex, time-dependent processes underlying conception success (20). Molecular profiling offers a path to overcoming this gap by revealing latent biological signatures that precede and potentially predict fertility outcomes.

Such latent biological signatures are especially likely to be detectable in reproductive tract secretions, which directly reflect the local physiological state during the peri-conception period. Among cow reproductive secretions, cervical mucus (CM) plays a pivotal gatekeeping role in sperm transport, survival and fertilisation. Its protein composition is dynamically modulated by endocrine cues across the peri-ovulatory window, and includes mucins, innate-immune mediators, chaperones and enzymes implicated in microenvironmental remodelling (9). These features position the CM proteome as a biologically plausible source of fertility-linked biomarkers that could be exploited in practical screening tools in the field.

While proteomics has advanced rapidly in male reproductive biology (*e.g*. in analysing seminal plasma and spermatozoa), comprehensive studies of female tract secretions, and particularly buffalo CM, remain scarce, and data from Indonesian swamp buffalo populations are virtually non-existent (8). This evidentiary gap limits both mechanistic understanding and the development of marker panels tailored to local breeds and production contexts.

The present study directly addresses this gap. As a follow-up to earlier work in the same population and field context, high-resolution liquid chromatography–tandem mass spectrometry (LC–MS/MS) was applied to characterise the cervical-mucus proteome of swamp buffalo heifers in a study focused exclusively on discovery-scale proteomics without reintroducing non-proteomic scoring frameworks. Protein identification and label-free quantification were undertaken, followed by statistical prioritisation of candidate markers and functional annotation to delineate pathways potentially underpinning successful fertilisation. By providing the first systematic CM proteome map in Indonesian swamp buffalo, this study establishes a molecular reference for selection for fertility-and for AI management in tropical smallholdings (1).

## Material and Methods

### Study design and ethics

This was a label-free discovery proteomics investigation of CM from swamp buffalo (*Bubalus bubalis*) and followed up prior work in the same population and field context, extending the scope toward proteome mapping for protein biomarker discovery. Field sampling was conducted in South Sulawesi, Indonesia, between May and November 2024. Initial sample handling occurred at a university animal reproduction laboratory, and LC–MS/MS acquisition was performed at a national proteomics core facility. All procedures complied with institutional animal welfare guidelines and were approved by the relevant animal ethics committee (Approval No. 093/KE.02/SK/05/2023).

### Animals and sample collection

Forty healthy nulliparous swamp buffalo heifers, approximately 2–5 years old, were enrolled. Cervical mucus was collected aseptically 5–30 min before the first AI during oestrus by trained personnel using a sterile speculum and single-use catheter. Samples were transferred into nuclease- and protease-free tubes, snap-frozen in liquid nitrogen and stored at −80°C until analysis. To ensure comparability with an earlier study, the timing and handling of sampling were aligned to the previously described methodological framework without re-introducing non-proteomic assessments (22).

### Protein extraction and in-solution digestion

Frozen aliquots were thawed on ice, briefly clarified by low-speed centrifugation, and subjected to buffer exchange into 50 mM ammonium bicarbonate to mitigate matrix effects from mucins and salts. Proteins were solubilised in 8 M urea with protease inhibitors. Disulphide bonds were reduced with 10 mM tris(2-carboxyethyl)phosphine at room temperature for 30 min and alkylated with 20 mM iodoacetamide for 30 min in the dark. The urea concentration was then diluted to below 1.5 M prior to proteolysis with a trypsin/lys-C mixture at an enzyme-to-substrate ratio of 1:50 and incubated at 37°C for 12–16 h. The resulting peptides were cleaned on C18 spin devices, eluted with 70% acetonitrile containing 0.1% formic acid, vacuum-dried and reconstituted in 2% (v/v) acetonitrile with 0.1% (v/v) formic acid for LC–MS/MS injection (14).

### NanoLC–MS/MS acquisition

Peptides were analysed on a NanoLC Ultimate 3000 system coupled to a Q Exactive Plus Orbitrap mass spectrometer (Thermo Scientific, Sunnyvale, CA, USA) equipped with a nano-C18 column (75-μm inner diameter, 25-cm length, with 1.6–2.0 μm particles; Thermo Scientific, Sunnyvale, CA, USA). The flow rate was 300 nL min^−1^ under a 60–120-min gradient comprising 5–30% solvent B, 30–45% B, and a terminal wash at 80% B. Solvent A was water with 0.1% formic acid and solvent B acetonitrile with 0.1% formic acid. Ionisation was performed in positive electrospray ionisation mode. Full-scan mass spectra were acquired at 70,000 resolution (m/z 200), across m/z 350–2,000, with an automatic gain control target of 3 × 10^6^ and a maximum injection time of 50 ms. Data-dependent acquisition selected the top 15 precursors for higher-energy collisional dissociation, and fragment-ion spectra were acquired at 17,500 resolution, automatic gain control target of 1 × 10^5^, maximum injection time 60 ms, normalised collision energy 27–30%, isolation width 1.6 m/z and dynamic exclusion of 30–45 s (2).

### Database and protein identification

Raw data were processed in Proteome Discoverer v2.2 (Thermo Fisher Scientific, USA) and searched against the latest UniProt *Bubalus bubalis* reference database supplemented with common contaminants and a decoy set; a *Bos taurus* supplement was included to enhance cross-species annotation where appropriate. Searches assumed trypsin/P specificity with up to two missed cleavages, carbamidomethylation of cysteine as a fixed modification and methionine oxidation and N-terminal acetylation as variable modifications. False discovery was controlled by a target–decoy approach with peptide- and protein-level false detection rate <1%. Only proteins identified with at least two unique peptides were advanced to quantitative analyses (24).

### Label-free quantification, missing-data handling, and quality control

Label-free quantification was performed using extracted ion chromatographic peak areas. Peptide features were detected and aligned with the Minora algorithm, and protein abundances were inferred with the MaxLFQ (label-free quantification) algorithm in Proteome Discoverer followed by log_10_ transformation and inter-sample normalisation using total ion current or locally estimated scatterplot smoothing to reduce systematic variation. Missing intensities were treated as left-censored values and imputed with distribution-aware minimal-value strategies suitable for discovery proteomics. Quality control comprised pooled-QC injections at regular intervals (every 5–10 runs) targeting QC LFQ coefficients of variation <20%, indexed retention time peptide standards (iRT) for retention-time calibration, and technical replicates on a subset of samples. Post-normalisation data integrity and outlier detection were assessed by principal component analysis and distance-to-centre metrics (11).

### Statistical and bioinformatics analyses

The analyses were performed in R/Python. Distributional assumptions were examined using Shapiro–Wilk tests for normality and Levene’s test for homoscedasticity. Between-group differential abundance, defined on biologically relevant endpoints without reusing non-proteomic scores, was evaluated using moderated linear models (limma) or parametric/nonparametric tests as appropriate, with Benjamini–Hochberg correction for multiple testing. Features were deemed significant at q (the false-discovery-rate-adjusted P-value) <0.05 in conjunction with pre-specified log_2_ fold-change thresholds. Global structure and separation were visualised with score plots (principal component analysis (PCA) and partial least-squares discriminant analysis (PLS-DA) accompanied by permutation testing), clustered heatmaps, Venn summaries after QC filtering and volcano plots. Discriminative performance of single-marker and panel candidates was estimated by ROC analysis reporting AUC, Youden’s J–optimised cut-offs, sensitivity, and specificity under stratified k (number of data partitions)-fold cross-validation (*e.g*. k = 5) to mitigate overfitting (4).

### Functional annotation and targeted verification

**Fig. 1.**
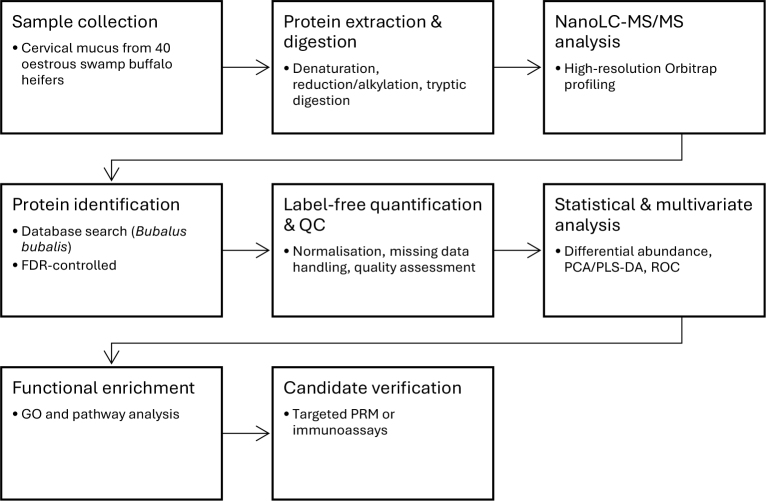
Schematic workflow of cervical-mucus proteomic biomarker discovery in swamp buffalo. LC-MS/MS – liquid chromatography with tandem mass spectrometry; FDR – false detection rate; PCA/PLS-DA – principal component analysis and partial least-squares discriminant analysis; GO – Gene Ontology database; PRM – parallel reaction monitoring

Direction-specific protein sets underwent enrichment analysis using the g:GOSt module within the g:Profiler functional enrichment identification tool. The analysis was made across the Biological Process, Molecular Function and Cellular Component domains of the Gene Ontology functional annotation database, the Kyoto Encyclopedia of Genes and Genomes and the Reactome pathway database where available, applying multiple-testing adjustment and reporting terms with adjusted P-value < 0.05 alongside leading proteins and concise biological interpretations. Top candidates were subsequently verified in a targeted manner. Verification comprised parallel and selected reaction monitoring (PRM and SRM) on proteotypic peptides (at least two per protein) or by ELISA/Western blot when antibodies were available. Diagnostic performance was summarised on a separate run batch and, where feasible, an independent subset (10). An overview of the experimental and analytical workflow is provided in Fig. 1.

## Results

### Protein identification and distribution

**Fig. 2.**
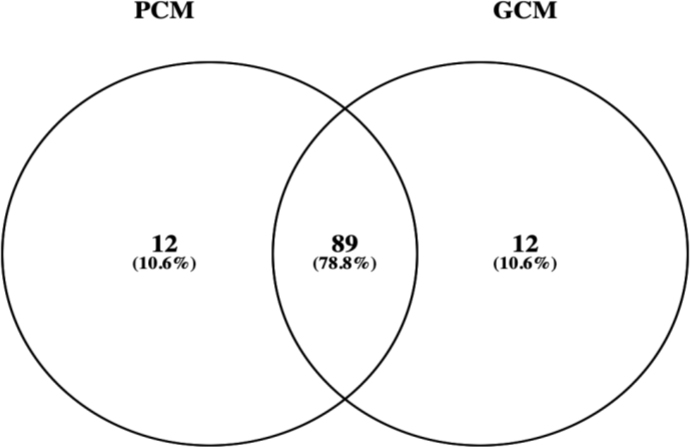
Venn diagram of retained features after QC (good swamp-buffalo cervical mucus (GCM) vs poor cervical mucus (PCM))

As shown in Fig. 2, quality control retained 113 protein features in total: 89 (78.8%) were shared between groups, while 12 (10.6%) were unique to good cervical mucus (GCM) and 12 (10.6%) were unique to poor cervical mucus (PCM). Accordingly, the post-QC set sizes were GCM = 101 and PCM = 101. This large shared core indicates a broadly conserved cervical-mucus proteome across groups, with only modest group-specific tails

### Differential protein profile

**Fig. 3.**
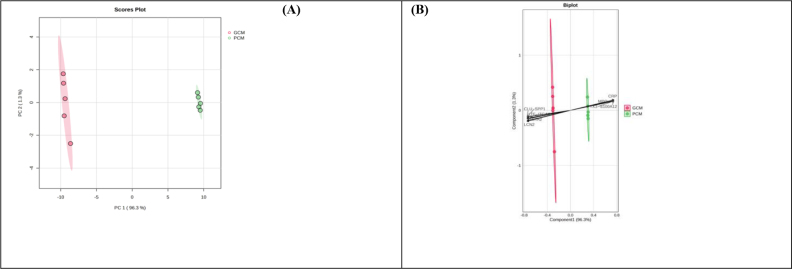
Principal component analysis of swamp-buffalo cervical mucus proteins. (A) Score plot of PC1 *vs* PC2 showing group separation with 95% confidence ellipses. (B) Biplot illustrating the contribution of selected proteins to group separation. GCM – good cervical mucus; PCM – poor cervical mucus; CLU– clusterin; SPP1 – osteopontin; LTF – lactotransferrin; SLPI – secretory leukocyte protease inhibitor; LCN2 – lipocalin-2; MPO – myeloperoxidase; PTX3 – pentraxin 3; CRP – C-reactive protein; S100A12 – S100 calcium-binding protein A12

As shown in Fig. 3, PCA revealed a clear, group-wise separation driven predominantly along PC1 (capturing the vast majority of total variance), with only modest dispersion on PC2. The PC1–PC2 scores plot (Fig. 3B) shows two compact, non-overlapping clusters for GCM and PCM of which the 95% confidence ellipses do not intersect, indicating strong between-group structure relative to within-group variation. The accompanying biplot (3B) highlights a subset of proteins with high absolute loadings on PC1 that oppose in sign across groups, consistent with systematic quantitative shifts rather than idiosyncratic sample noise. Collectively, these patterns support a robust global proteomic distinction between GCM and PCM.

**Fig. 4.**
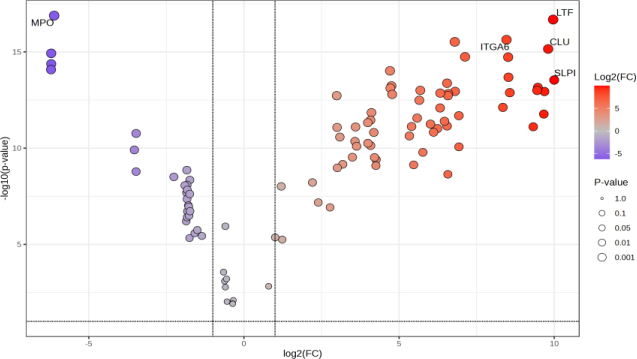
Volcano plot of differential proteins in good swamp-buffalo cervical mucus and in poor cervical mucus. FC – fold change; MPO – myeloperoxidase; ITGA6 – integrin α6; LTF – lactotransferrin; CLU – clusterin; SLPI – secretory leukocyte protease inhibitor

**Fig. 5.**
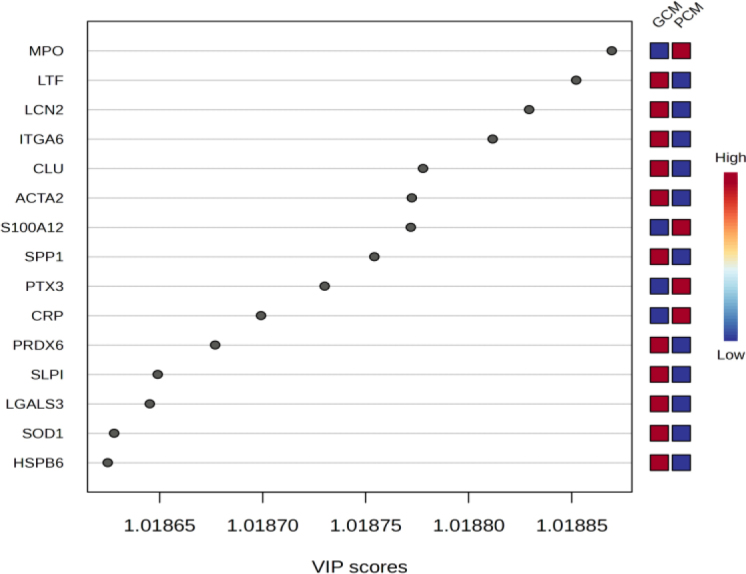
Partial least-squares discriminant analysis variable-importance (VIP) scores for the top 15 proteins in swamp-buffalo cervical mucus. GCM – good cervical mucus; PCM – poor cervical mucus; MPO – myeloperoxidase; LTF – lactotransferrin; LCN2 – lipocalin-2; ITGA6 – integrin α6; CLU – clusterin; ACTA2 – actin α2, smooth muscle; S100A12 – S100 calcium-binding protein A12; SPP1 – osteopontin; PTX3 – pentraxin 3; CRP – C-reactive protein; PRDX6 – peroxiredoxin 6; SLPI – secretory leukocyte protease inhibitor; LGALS3 – galectin-3; SOD1 – superoxide dismutase 1; HSPB6 – heat-shock protein B6

As shown in Fig. 4, volcano analysis revealed a strongly asymmetric pattern favouring GCM, with numerous high-magnitude, statistically supported increases in proteins involved in epithelial integrity and mucosal defence. Among the most upregulated in GCM were lactotransferrin (LTF), integrin a6 (ITGA6), clusterin (CLU), lipocalin-2 (LCN2), secretory leukocyte protease inhibitor (SLPI), osteopontin (SPP1), peroxiredoxin-6 (PRDX6), heat-shock proteins B6 and B1(HSPB6/HSPB1), transthyretin, vitronectin (VTN), fibronectin (FN1), and thrombospondin-2 (THBS2). In contrast, proteins enriched in PCM clustered on the negative side and were led by myeloperoxidase (MPO), S100 calcium-binding protein A12, pentraxin-3 and C-reactive protein (CRP), consistent with heightened neutrophil and acute-phase signalling. Table 1 corroborates these shifts with large effect sizes and raw P-values spanning approximately 10^−9^ to 10^−13^. Consistent with these differential-abundance results, PLS-DA variable-importance (VIP) analysis (Fig. 5) converges on the same markers MPO and LTF at the extremes, while also prioritising LCN2, ITGA6, CLU, actin alpha 2 smooth muscle (ACTA2), SPP1, CRP, PRDX6, SLPI, galectin 3 (LGALS3) and SOD1 as key contributors to group separation. Taken together, these results indicate that GCM is characterised by an enriched proteome having barrier, adhesion and antioxidant functions, whereas PCM exhibits a signature dominated by neutrophil-derived and acute-phase proteins.

**Table 1.** Proteins expressed differentially between good cervical mucus (GCM) and poor cervical mucus (PCM) from swamp buffalo

Protein	FC	Log_2_ (FC)	P-value	Direction
MPO	1.44 × 10^−2^	-6.117	1.31 × 10^−13^	PCM
LTF	4.17 × 10^1^	5.381	2.09 × 10^−13^	GCM
ITGA6	3.53 × 10^2^	8.462	2.33 × 10^−12^	GCM
LCN2	4.63	2.211	3.00 × 10^−12^	GCM
CLU	8.94 × 10^2^	9.804	7.05 × 10^−12^	GCM
S100A12	1.35 × 10^−2^	-6.212	1.19 × 10^−11^	PCM
ACTA2	5.83	2.544	1.81 × 10^−11^	GCM
SPP1	1.53 × 10^1^	3.931	1.88 × 10^−11^	GCM
PTX3	1.35 × 10^−2^	-6.211	4.19 × 10^−11^	PCM
CRP	1.34 × 10^−2^	-6.217	8.29 × 10^−11^	PCM
PRDX6	2.61 × 10^4^	14.673	9.64 × 10^−11^	GCM
ITGB1	1.54 × 10^1^	3.942	2.08 × 10^−10^	GCM
SLPI	4.26 × 10^1^	5.414	2.89 × 10^−11^	GCM
LGALS3	9.35 × 10^4^	16.512	4.22 × 10^−10^	GCM
SOD1	2.70 × 10^4^	14.721	5.82 × 10^−10^	GCM
HSPB6	7.10 × 10^2^	9.472	6.99 × 10^−10^	GCM
ANXA5	2.63 × 10^4^	14.685	7.67 × 10^−11^	GCM
bTrappin	2.15	1.107	9.96 × 10^−10^	GCM
TTR	6.99 × 10^2^	9.449	1.00 × 10^−9^	GCM
VTN	4.63	2.211	1.13 × 10^−9^	GCM
HSPB1	3.43 × 10^1^	5.101	1.14 × 10^−9^	GCM
FN1	3.80 × 10^2^	8.569	1.31 × 10^−9^	GCM
COL3A1	8.03 × 10^4^	16.293	1.38 × 10^−9^	GCM
THBS2	9.60 × 10^4^	16.551	1.48 × 10^−9^	GCM
GPX1	2.74 × 10^4^	14.744	1.61 × 10^−9^	GCM
ACTA1	9.54 × 10^4^	16.541	1.87 × 10^−9^	GCM

1 FC – fold change; MPO – myeloperoxidase; LTF – lactotransferrin; ITGA6 – integrin α6; LCN2 – lipocalin-2; CLU – clusterin; S100A12 – S100 calcium-binding protein A12; ACTA2 – actin α2, smooth muscle; SPP1 – osteopontin; PTX3 – pentraxin 3; CRP – C-reactive protein; PRDX6 – peroxiredoxin 6; ITGB1 – integrin β1: SLPI – secretory leukocyte protease inhibitor; LGALS3 – galectin-3; SOD1 – superoxide dismutase 1; HSPB6 – heat-shock protein B6; ANXA5 – annexin A5; bTrappin – trappin-family whey acidic protein-domain protease inhibitor; TTR – transthyretin; VTN – vitronectin; HSPB1 – heat-shock protein B1; FN1 – fibronectin 1; COL3A1 – collagen type 3 α1 chain; THBS2 – thrombospondin 2; GPX1 – glutathione peroxidase 1; ACTA1 – actin α1, skeletal muscle

### Functional enrichment

**Fig. 6.**
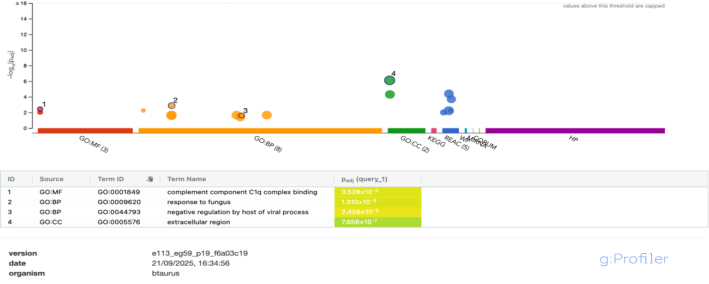
Functional enrichment of direction-specific protein sets in swamp-buffalo cervical mucus. GO:MF – Molecular Function in the Gene Ontology database; GO:BP – Biological Process in the Gene Ontology database; GO:CC – Cellular Component in the Gene Ontology database; KEGG – Kyoto Encyclopedia of Genes and Genomes; REAC – Reactome pathway database; WP – WikiPathways; MIRNA – microRNA-related pathways; CORUM – Comprehensive Resource of Mammalian Protein Complexes

As shown in Fig. 6, enrichment analysis emphasised secreted innate-immunity signatures within the cervical-mucus proteome. The broad “extracellular region” term was highly significant (adjusted P-value = 7.66 × 10^−7^) and encompassed the major discriminants identified earlier, including GCM-leaning LTF, LCN2, SPP1, SLPI, LGALS3 and HSPB6 together with PCM-leaning MPO, PTX3 and CRP. This was consistent with a predominantly exocrine and secretory signature (Table 2). Host-defence terms were selectively driven by PCM-enriched markers: “response to fungus” (MPO and PTX3; adjusted P-value = 1.31×10^−3^) and “complement component C1q complex binding” (PTX3 and CRP; adjusted P-value = 3.53 × 10^−3^). A separate enrichment of the “negative regulation by host of viral process” marker was observed (PTX3; adjusted P-value = 2.46 × 10^−2^). Together these results indicate a functional dichotomy whereby PCM is characterised by augmented acutephase, complement and neutrophil-associated activity, whereas GCM shows a secreted barrier and antimicrobial profile without overt inflammatory skewing, reinforcing the quantitative patterns observed in the volcano and VIP analyses.

**Table 2.** Functional annotation of selected proteins and implications for swamp-buffalo cervical mucus quality

Source	Term ID	Term name	Adjusted P-value	Protein
GO:MF	GO:0001849	complement component C1q complex binding	3.528 × 10-3	PTX3; CRP
GO:BP	GO:0009620	response to fungus	1.310 × 10-3	MPO; PTX3
GO:BP	GO:0044793	negative regulation by host of viral process	2.456 × 10-2	PTX3
GO:CC	GO:0005576	extracellular region	7.656 × 10-7	MPO; LTF; LCN2; SPP1; PTX3; CRP; SLPI; LGALS3; HSPB6

1 GO:MF – Molecular Function in the Gene Ontology database; GO:BP – Biological Process in the Gene Ontology database; GO:CC – Cellular Component in the Gene Ontology database; PTX3 – pentraxin 3; CRP – C-reactive protein; MPO – myeloperoxidase; LTF – lactotransferrin; LCN2 – lipocalin-2; SPP1 – osteopontin; SLPI – secretory leukocyte protease inhibitor; LGALS3 – galectin-3; HSPB6 – heat-shock protein B6

### Biomarker analysis (ROC)

**Fig. 7.**
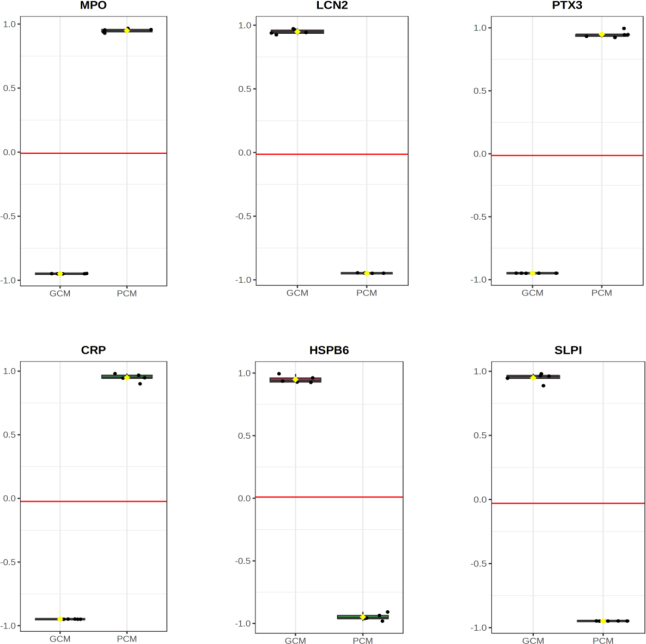
ROCs for candidate biomarkers in swamp-buffalo cervical mucus. GCM – good cervical mucus; PCM – poor cervical mucus; MPO – myeloperoxidase; LCN2 – lipocalin-2; PTX3 – pentraxin 3; CRP – C-reactive protein; HSPB6 – heat-shock protein B6; SLPI – secretory leukocyte protease inhibitor

Single-protein classification yielded complete separation for six candidates in this cohort. Three markers elevated in GCM – LCN2, SLPI and HSPB6 – each achieved AUC = 1.00 with perfect sensitivity and specificity at the Youden’s J–optimised cut-offs (Fig. 7, Table 3), mirroring their large positive log_2_ FC and low P-values from the differential analysis. Likewise, three markers elevated in PCM – MPO, PTX3 and CRP – also produced AUC = 1.00 with non-overlapping distributions across groups. In contrast, additional candidates – LTF, SPP1 and LGALS3 – showed strong but non-perfect discrimination (AUC = 0.86, 0.86 and 0.84, respectively). Taken together, these results nominate LCN2, SLPI and HSPB6 as leading single-analyte classifiers in GCM and MPO, PTX3 and CRP as the same in PCM, supported by concordant directions in the volcano plot and inclusion among VIP >1 features.

**Table 3.** Classification performance of biomarker candidates

Protein	Direction	AUC
MPO	PCM	1
LTF	GCM	0.86
LCN2	GCM	1
SPP1	GCM	0.86
PTX3	PCM	1
CRP	PCM	1
SLPI	GCM	1
LGALS3	GCM	0.84
HSPB6	GCM	1

1 PCM – poor cervical mucus; GCM – good cervical mucus; MPO – myeloperoxidase; LTF – lactotransferrin; LCN2 – lipocalin-2; SPP1 – osteopontin; PTX3 – pentraxin 3; CRP – C-reactive protein; SLPI – secretory leukocyte protease inhibitor; LGALS3 – galectin-3; HSPB6 – heatshock protein B6

## Discussion

This discovery-scale map of the swamp-buffalo cervical-mucus proteome builds directly on our previous study in the same population and field context, which established the sampling framework and clinical grouping used here and highlighted the need for molecular readouts to complement field selection (12). Consistently with that groundwork, we find that group differences are predominantly quantitative, not presence or absence: most features were shared post QC, yet unsupervised PCA still separated groups cleanly along the leading component (21). This pattern reinforces the earlier inference that peri-ovulatory mucosal biology, rather than wholesale proteome turnover, underlies fertility differences in this system, a conclusion that aligns with studies in cattle showing that subtle quantitative shifts in cervical and uterine secretions around oestrus are associated with conception success (6, 7).

Extending beyond the phenotypic signals reported earlier, the present proteomic analysis reveals a biological dichotomy: GCM is enriched in barrier and adhesion functions, antimicrobial proteins and antioxidant capacity, whereas PCM shows a proteomic axis associated with classical inflammatory markers, including neutrophil and acute-phase proteins (17). Marker-level results exemplify this: LCN2, SLPI and HSPB6 (GCM) met the VIP > 1 criterion, mapped to significant enrichment terms, and each achieved AUC = 1.00; MPO, PTX3 and CRP (PCM) met the same orthogonal criteria with perfect classification (16). Importantly, these proteins have been independently linked in cattle to reproductive tract conditions that influence fertility outcomes, including sperm survival, immune tolerance at insemination and successful establishment of pregnancy, providing context for the pattern shown in our findings (23).

Functionally, the strong enrichment for the extracellular region alongside host-defence terms (*e.g*. response to fungus and binding to the C1q-complex – the initiating component of the classical complement pathway) aligns with our prior proposal that secreted luminal factors at the cervix govern sperm survival and transport (15). In cattle, maintenance of a noninflammatory cervical environment during oestrus has been associated with higher conception rates and improved sperm passage. Within this framework, the coordinated abundance of proteins associated with adhesion and the extracellular matrix (ITGA6, FN1, THBS2 and VTN) alongside antioxidant enzymes (PRDX6, GPX1 and SOD1) in GCM provides molecular correlates of a receptive, barrier-competent mucosal milieu; such conditions are consistent with bovine studies linking epithelial integrity, oxidative balance and moderated immune activity to favourable fertility outcomes. Conversely, the PCM signature dominated by MPO, S100A12, PTX3 and CRP comprises proteins commonly associated with neutrophil-related, oxidative and complement-linked processes and noted in inflammation. This profile was repeatedly associated with reduced conception rates and impaired fertility in cattle when present around the time of insemination (16).

At the level of individual candidates, the biological roles of the identified markers further reinforce their relevance to fertility. Lipocalin 2 and SLPI are known modulators of mucosal immunity, limiting excessive neutrophil activation while preserving antimicrobial protection; elevated expression of these proteins in the bovine reproductive tract has been associated with improved sperm viability and reduced inflammatory damage. Heat-shock protein B6 participates in cellular stress responses and smooth muscle regulation, processes relevant to cervical receptivity and sperm transport. In contrast, elevated MPO, PTX3 and CRP reflect acute inflammatory responses and neutrophil activation, conditions that have been linked to suboptimal fertilisation and early reproductive failure in cattle. Together, these associations provide a mechanistic bridge between the proteomic signatures observed here and established fertility outcomes in bovine species (7).

The six high-confidence proteins – LCN2, SLPI and HSPB6 in GCM and MPO, PTX3 and CRP in PCM – now constitute testable biomarkers for targeted verification by PRM/SRM or ELISA, either individually or as multi-analyte panels. The panels would be constituted as noted, with cut-offs derived from Youden’s J as demonstrated by Fair *et al*. (3). Such panels directly address the previously highlighted limitations of subjective selection and heterogeneity in field detection by anchoring decisions to reproducible molecular readouts with known links to fertility outcomes in cattle (25).

Some limitations of this study are apparent: the discovery-scale sample size may inflate apparent AUC values, label-free quantification is sensitive to missingness and batch effects, and cross-species database supplementation may influence protein inference in *Bubalus bubalis* (13). Accordingly, we echo our earlier recommendation for independent cohort validation explicitly linked to conception outcomes, finer peri-ovulatory time-course sampling and development of field-ready assays with stability testing across rural cold-chain conditions (19).

In summary, this work defines coherent extracellular proteomic signatures that differentiate good-from poor-quality cervical mucus, and suggests LCN2, SLPI and HSPB6 as a set and MPO, PTX3 and CRP as another set of biologically plausible, high-performing biomarkers with established relevance to fertility outcomes in cattle. The results provide a mechanistic and translational foundation for molecularly informed fertility selection in swamp buffalo AI programmes, and suggest a path from discovery to validation and deployment.

## Conclusion

This follow-up study indicates that the cervical-mucus proteome of swamp buffalo exists in two biologically distinct states: one consistent with receptivity and barrier-competence, characterised by adhesion and ECM interactors, antimicrobial effectors and cytoprotective stress responses; and the other state dominated by neutrophil- and acute-phase proteins with prominent complement engagement and oxidative processes. Within this framework, LCN2, SLPI and HSPB6 emerge as hallmarks of mucosal defence and epithelial resilience, whereas MPO, PTX3 and CRP reflect a heightened innate-immune response likely unfavourable to sperm survival and transport. These mechanistic signatures provide a coherent explanation for fertility differences at the cervix and nominate a practical set of secreted protein biomarkers for further evaluation oftheir suitability for translation into targeted assays and field screening. By linking female selection for fertility to molecular pathways in the reproductive tract rather than to external phenotype alone, this study may improve AI outcomes in tropical smallholder systems. Ifits findings are reproduced, this research may guide subsequent assay development and validation in independent herds.
